# Effects of women’s footwear on the mechanical function of heel-height accommodating prosthetic feet

**DOI:** 10.1371/journal.pone.0262910

**Published:** 2022-01-24

**Authors:** Matthew J. Major, Julia Quinlan, Andrew H. Hansen, Elizabeth Russell Esposito

**Affiliations:** 1 Department of Physical Medicine & Rehabilitation, Northwestern University, Chicago, IL, United States of America; 2 Department of Biomedical Engineering, Northwestern University, Evanston, IL, United States of America; 3 Jesse Brown VA Medical Center, Chicago, IL, United States of America; 4 Minneapolis VA Health Care System, Minneapolis, MN, United States of America; 5 University of Minnesota, Minneapolis, MN, United States of America; 6 DoD-VA Extremity Trauma and Amputation Center of Excellence (EACE), San Antonio, TX, United States of America; 7 Center for Limb Loss and Mobility, VA Puget Sound Health Care System, Seattle, WA, United States of America; 8 University of Washington, Seattle, WA, United States of America; 9 Uniformed Services University of the Health Sciences, Bethesda, MD, United States of America; West Park Healthcare Centre, CANADA

## Abstract

The loaded mechanical function of transtibial prostheses that result from the clinical assembly, tuning, and alignment of modular prosthetic components can directly influence an end user’s biomechanics and overall mobility. Footwear is known to affect prosthesis mechanical properties, and while the options of footwear are limited for most commercial feet due to their fixed geometry, there exists a selection of commercial prosthetic feet that can accommodate a moderate rise in heel height. These feet are particularly relevant to women prosthesis users who often desire to don footwear spanning a range of heel heights. The aim of this study was to assess the effects of adding women’s footwear (flat, trainer, 5.08 cm heel) on the mechanical properties (deformation and energy efficiency) of four models of heel-height accommodating prosthetic feet. Properties were measured through loading-unloading at simulated initial contact, midstance and terminal stance orientations with a universal materials test system, and statistically compared to a barefoot condition. Results suggest that the addition of footwear can alter the level of foot deformation under load, which may be a function of the shoe and alignment. Moreover, while each foot displayed different amounts of energy storage and return, the addition of footwear yielded similar levels of energy efficiency across foot models. Overall, prosthesis users who don shoes of varying heel heights onto adjustable prosthetic feet and their treating clinicians should be aware of the potential changes in mechanical function that could affect the user experience.

## Introduction

There is a growing body of evidence to suggest that the stance-phase mechanical properties of prosthetic feet (stiffness, damping, roll-over geometry) have a direct impact on clinically-relevant user performance outcomes, including stability, metabolic cost, whole-body dynamics, and residual limb loading [1, 2]. Moreover, prosthesis users can detect small differences in these properties and express preference for foot function during certain walking scenarios [3, 4]. Consequently, it is important for prosthetists to determine the optimal prosthesis through: 1) careful selection and mechanical tuning of prosthetic componentry based on manufacturer recommendations according to user characteristics and activity level, and 2) subsequent alignment adjustments to satisfy clinical and individual patient user objectives [1]. The mechanical properties of the prosthetic foot have well-recognized effects on the abilities of the end user and how they move [5, 6], but prosthetists have very limited access to the particular mechanical function of different prosthetic feet [7]. The clinical design process is reliant on a combination of clinical experience, available guidelines, and clinical objectives shared between the rehabilitation team and patient.

In addition to the mechanical properties of the prosthetic foot, other factors also influence the mechanical function. For example, although prosthetists clinically optimize the definitive prosthesis as a function of the combined mechanical properties resulting from assembled components, they have little control over the types of footwear used outside the clinic, which can considerably affect those properties [8]. For most commercial prosthetic feet, the selection of footwear is rather limited as these devices have a fixed heel-to-toe height differential. However, there is a small selection of commercially available prosthetic foot designs that are specifically designed to accommodate heel heights of up to 5.08 cm, either by adjusting the pylon-to-foot angle through an ankle articulation that can be controlled by the user, or by swapping prosthetic feet of different but fixed geometries [9, 10]. These prosthetic feet are designed to accommodate footwear that are often worn by women. The desire of women to wear a range of shoes reflects their need to select footwear that matches attire for a specific occasion, thereby facilitating community participation. While the unique needs and challenges of women with limb loss have been recognized, women prosthesis users have received notably less attention in prosthetics research [9, 11]. Consequently, women-specific prosthetics research is imperative to improving overall evidence-based clinical practice.

To date, there is limited information on the effects of women-specific footwear with prosthetic feet that can accommodate different heel rises/heights [12, 13], and particularly the user-independent stance-phase mechanical properties [12]. For those prosthetic feet that can be adjusted through an ankle articulation, their change in mechanical properties is a function of both added footwear and changes in alignment. Characterizing the mechanical function of these prosthesis-shoe combinations will add to the important body of knowledge on prosthetic solutions for women with lower limb loss. With such information, prosthetists can more confidently make informed decisions on selecting components for women prosthesis users and educate their patients on the potential impact of swapping shoes. Importantly, reporting the mechanical properties of lower limb prosthetic components will allow the field to classify feet based on their quantified function rather than categorizing them nominally by type, a practice that lacks standardization in the field. Therefore, the aim of this study was to quantify the effects of women-specific footwear on the mechanical properties of commercially available prosthetic feet that accommodate heel heights up to 5.08 cm. Given previous findings on footwear effects with prosthetic feet [8, 14], we hypothesized that the addition of footwear would alter prosthesis mechanical properties, specifically by 1) increasing deformation and 2) decreasing energy return (i.e., energy efficiency) relative to a barefoot condition without a shoe.

## Methods

Four commercial prosthetic feet were assessed in this study: three heel-height adjustable prostheses and a series of Solid Ankle Cushion Heel (SACH) feet that were recommended by manufacturers for a 68–70 kg patient (right side, 24 cm foot length), but with mass limits exceeding 100 kg. The adjustable feet allowed for changes in sagittal-plane ankle angle (foot position relative to shank) through an articulated joint either activated by depressing a locking button or controlled through a wrench to accommodate heel height while allowing a shank pylon to remain at some set angle (e.g. vertical). These feet included the Runway (Category 4, adjustable heel height 0–5.08 cm, Freedom Innovations LLC, Irvine, CA), Pro-Flex Align (Category 3, adjustable heel height 0–7 cm, Össur Americas, Foothill Ranch, CA), and Accent (Category 3, adjustable heel height 0–5.08 cm, College Park Industries, Warren, MI), all rated for moderate impact activity. The SACH feet (Kingsley Mfg. Co., Costa Mesa, CA) were at a fixed geometry and so three feet (0, 2.54, and 4.45 cm heel rise), were used to accommodate the different heel heights, all of which were of firm stiffness category. To our knowledge, these feet represent nearly all of the currently available commercial options for non-microprocessor controlled feet that can be aligned by the user to accommodate moderate changes in footwear heel height.

**Fig 1:**
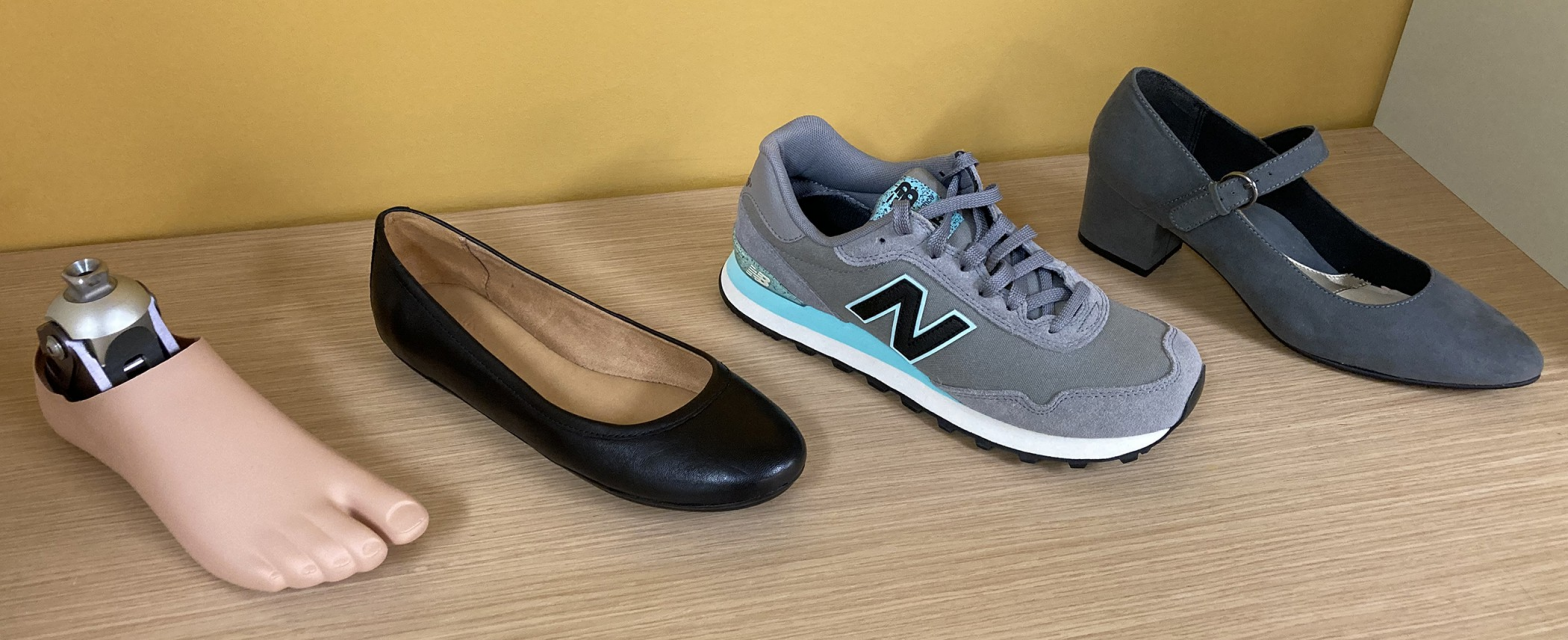
Four footwear conditions. Footwear heel height increases from left to right. Prosthesis and shoe images are similar but not identical to the original images and are therefore for illustrative purposes only.

**Fig 2:**
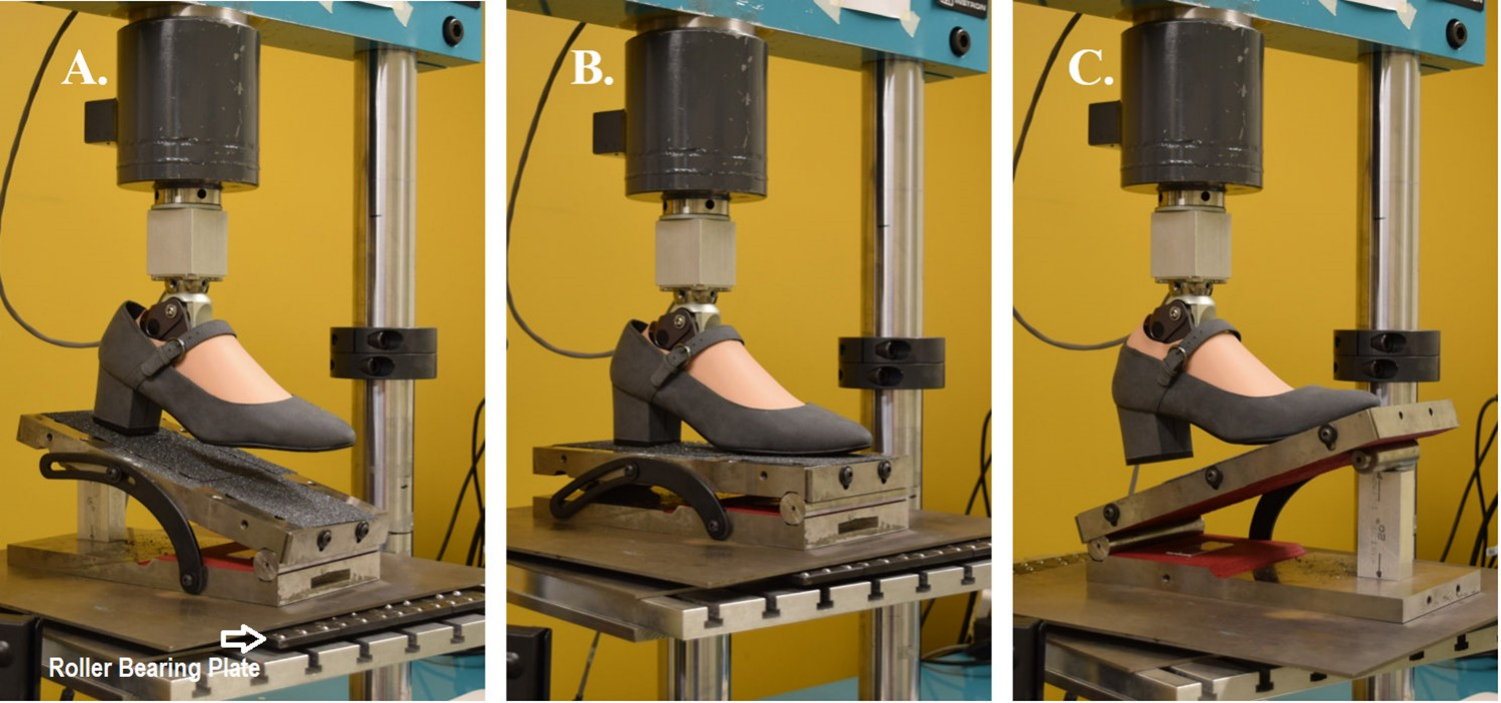
Test set up for each condition. A. Initial contact; B. midstance; C. terminal stance. Prosthesis and shoe images are similar but not identical to the original images and are therefore for illustrative purposes only.

**Fig 3:**
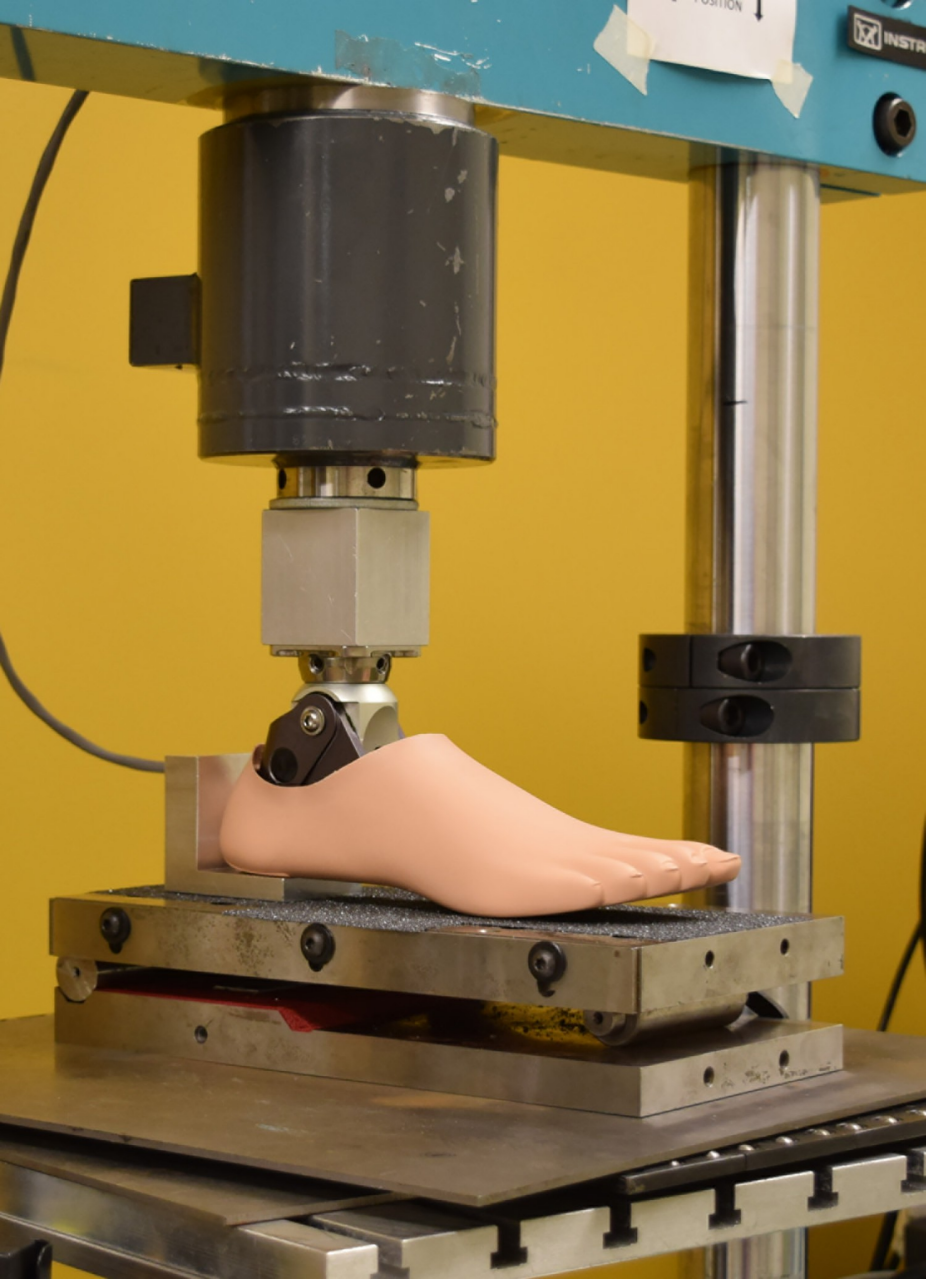
Test set up for the barefoot midstance condition, including the heel (L-)block.

Tests were conducted using a hydraulic-driven uniaxial materials testing system (Instron, Model 8800, Norwood, MA, USA) to measure instantaneous force and displacement. Prosthetic feet were tested barefoot (i.e., unshod) and under three heel height footwear conditions (**Fig 1**): 0-cm heel rise flat (Brittany, Naturalizer, St. Louis, MO); 3.18 cm heel rise trainer (Lifestyle 515, New Balance, Boston, MA); and 5.08 cm heel rise heeled dress shoe (referred to as the “heel” shoe) (Dustie, Soft Style, Richmond, IN). To note, the SACH 0, 2.54, and 4.45 cm rise versions were used to test the barefoot/flat, 3.18 cm trainer, and 5.08 cm heel, respectively. Shoes with varying heel heights were selected to serve as a range of footwear examples that women prosthesis users may prefer to use during activities of daily living and different social occasions [15]. In terms of distinguishing features, the flat shoe had minimal material, the trainer had a soft foam sole, and the 5.08 cm heel had a wide rigid heel. Prostheses-shoe combinations were loaded using three scenarios that reflect three critical instances during stance: initial contact (15° sagittal declined surface), midstance (level surface), and terminal stance (20° sagittal inclined surface) (**Fig 2**) [8, 16, 17]. For all cases, the prosthesis was aligned within the test system to a level loading surface (midstance, Fig 2B), secured through the proximal pyramid adapter, and the system proportional–integral–derivative control parameters were then tuned [8]. This alignment method was specific to each prosthesis-shoe setup and aided in setting the adjustable ankle angle for a given combination that was held constant across the three loading scenarios. The sine-plate for loading surface angle adjustments rested on roller bearings to minimize shear forces. A heel (L-)block of 1 cm height was placed under the heel end of the prosthesis for the barefoot midstance condition to account for plantar sole geometries with a heel rise (Fig 3). Since the 0-cm rise SACH foot was not designed with a small heel rise (toe-heel differential of approximately 1 cm) in the plantar surface geometry common to the adjustable ankle feet, this prosthesis was tested with and without the L-block for the barefoot midstance condition. For each footwear and loading condition, the prosthetic foot was pre-loaded to 100 N and then underwent two cycles of loading to 1230 N and unloading to 100 N at a loading rate of 200 N/s. Data from only the second load cycle were used for processing and this testing cycle was repeated five times. This loading regime was selected to remain consistent with previous methods [8]. While the manufacturer-recommended mass for these prostheses was slightly lower than the previous studies (i.e., 80 kg) [8, 16], to account for anthropometrics of women users, the applied maximum load still remained within the range that the prosthesis might experience when worn during ambulation.

Instantaneous force-displacement data were analyzed with custom MATLAB (MathWorks, Natick, MA) code to estimate two primary outcomes: maximum displacement (mm) and percent energy return (= energy return/energy stored×100%; %), and three secondary outcomes: energy stored (J), energy returned (J), and linear stiffness (linear best fit to the loading curve; N/mm). As prosthetic feet tend to possess non-linear loading behavior using these methods [8, 16], linear stiffness is presented only as a simple approximation of stiffness throughout the loading range. Maximum displacement provides information on the expected deformation of the prosthesis when loaded at each test orientation, while percent energy return is a measure of energy efficiency or energy dissipated due to damping as only a portion is returned when unloaded. Energy was calculated as the area under the force-displacement curve using the trapezoidal integration method. These outcomes were averaged over five trials for each prosthesis/shoe combination. While the variability of these bench test measurements are known to be small due to the deterministic nature of the observed mechanics [8, 16] and therefore may render statistical analysis unnecessary, statistical analyses were used to address the main hypotheses. The main effect of footwear (barefoot, flat, trainer, heel) on the primary outcomes of interest (maximum displacement, percent energy return) was assessed separately for each prosthesis and loading scenario using a one-way ANOVA (SPSS version 25, IBM, Armonk, NY), with post-hoc multiple comparisons performed with the Tukey HSD method to account for the Type I error rate. This post-hoc assessment allows us to address our hypothesis of the effect of wearing shoes compared to the unshod foot, but also compare each footwear condition against each other. For this analysis, there were 30 separate one-way ANOVAs conducted: 4 prostheses x 3 loading conditions x 2 outcomes, plus 3 loading conditions x 2 outcomes for the SACH foot when the L-block was included to align the barefoot condition. A secondary analysis of one-way ANOVAs (Tukey HSD post-hoc) was also conducted to assess the main effect of prosthesis for each footwear condition. A combination of Shapiro-Wilk tests and observation of Q-Q plots was used to identify violations of normality of the ANOVA model residuals. A follow-up Kruskal-Wallis test was used as a check if non-normality was suspected to engender confidence in the ANOVA results and if the result aligned with the one-way ANOVA the parametric analysis was retained. The critical α was set at 0.05 for all analyses.

## Results and discussion

**Fig 4:**
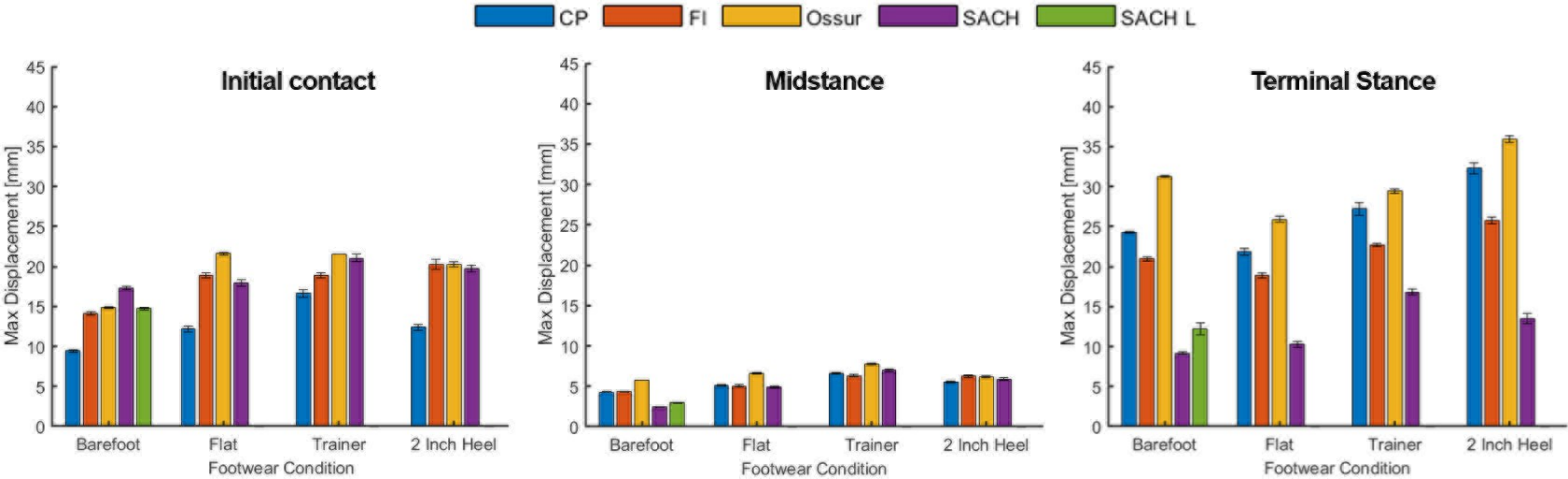
Maximum displacement of each prosthesis-footwear combination at initial contact, midstance, and terminal stance loading. CP = College Park; FI = Freedom Innovations; SACH L = barefoot condition with L-block. Error bars denote 95% confidence interval.

**Fig 5:**
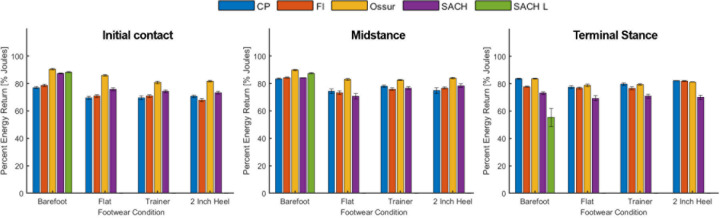
Percent energy return of each prosthesis-footwear combination at initial contact, midstance, and terminal stance loading. CP = College Park; FI = Freedom Innovations; SACH L = barefoot condition with L-block. Error bars denote 95% confidence interval.

Maximum displacement and percent energy return for each prosthesis-footwear combination and loading condition are presented in Figs 4 and 5, respectively. Stored and returned energy relative to barefoot (= shod condition value–barefoot condition value) are presented in Tables 1 and 2, respectively, and linear stiffness results are presented in Table 3. The results from the primary statistical analysis are displayed in Table 4, confirming that all conditions were significantly different than barefoot for each prosthesis and loading scenario, while not all shod conditions were different from each other. Table 5 displays results from the secondary statistical analysis. Absolute stored and returned energy (Joules), and representative force-displacement curves for each loading scenario and condition are presented in the S1 and S2 Tables, S1–S3 Figs. Raw instantaneous force-displacement data for each trial are available in a cloud-based digital repository (https://digitalhub.northwestern.edu/collections/769b817d-e4c5-4860-9e74-3077bd82b170).

**Table 1:** Energy stored relative to barefoot (Joules).

		Flat	Trainer	Heel
Initial contact	CP	1.5	4.1	1.6
	FI	3.2	3.0	3.1
	Össur	4.3	4.2	3.3
	SACH	1.3	1.9	1.6
Midstance	CP	0.3	1.3	0.6
	FI	0.5	1.2	1.0
	Össur	0.3	1.2	0.1
	SACH	1.4	2.7	2.2
Terminal stance	CP	-1.2	1.5	3.3
	FI	-2.0	-0.2	-0.2
	Össur	-4.2	-3.3	-2.4
	SACH	0.0	4.3	2.3

CP = College Park; FI = Freedom Innovations.

**Table 2:** Energy returned relative to barefoot (Joules).

		Flat	Trainer	Heel
Initial contact	CP	0.6	2.5	0.8
	FI	1.6	1.6	1.3
	Össur	3.4	2.6	2.0
	SACH	-0.2	0.2	-0.2
Midstance	CP	-0.1	0.8	0.2
	FI	0.1	0.7	0.6
	Össur	0.0	0.6	-0.2
	SACH	0.9	2.1	1.7
Terminal stance	CP	-1.8	0.6	2.4
	FI	-1.7	-0.4	0.3
	Össur	-4.2	-3.4	-2.4
	SACH	0.0	3.1	1.6

CP = College Park; FI = Freedom Innovations.

**Table 3:** Stiffness (N/mm; mean [95% confidence interval]).

		Barefoot	Flat	Trainer	Heel
Initial contact	CP	122.6 [0.7]	95.7 [1.5]	69.5 [1.1]	99.5 [1.7]
	FI	81.8 [0.6]	63.1 [0.5]	60.2 [0.5]	60.2 [1.4]
	Össur	72.2 [0.6]	52.7 [0.4]	53.2 [0.2]	59.7 [0.3]
	SACH	65.4 [0.4]	62.4 [0.9]	53.5 [0.8]	58.0 [0.8]
	SACH L	75.1 [0.5]	NA	NA	NA
Midstance	CP	266.1 [0.6]	223.9 [2.4]	175.0 [2.1]	209.8 [2.8]
	FI	266.4 [1.5]	228.2 [4.9]	183.6 [2.9]	182.8 [2.5]
	Össur	201.7 [1.1]	177.0 [2.0]	149.2 [1.4]	189.4 [2.4]
	SACH	475.1 [4.1]	227.2 [6.1]	163.7 [2.2]	191.1 [3.4]
	SACH L	417.4 [4.3]	NA	NA	NA
Terminal stance	CP	48.0 [0.2]	53.4 [0.9]	41.3 [0.6]	33.7 [0.5]
	FI	55.8 [0.7]	62.7 [0.6]	50.6 [0.5]	43.6 [0.4]
	Össur	38.4 [0.1]	44.6 [0.4]	37.5 [0.4]	26.7 [0.2]
	SACH	133.6 [1.8]	117.3 [3.6]	69.0 [1.1]	82.6 [3.0]
	SACH L	100.9 [6.9]	NA	NA	NA

CP = College Park; FI = Freedom Innovations; SACH L = barefoot condition with L-block.

**Table 4:** Statistical results to assess main effect of footwear.

	Prosthesis	F-value	p value	η2	Post-hoc					
					Bare	Bare	Bare	Flat	Flat	Trainer
					Flat	Trainer	Heel	Trainer	Heel	Heel
**Maximum Displacement**										
**Initial Contact**	CP	588.915	<0.001	0.991	<0.001	<0.001	<0.001	<0.001	0.651	<0.001
	FI	330.357	<0.001	0.984	<0.001	<0.001	<0.001	0.069	<0.001	0.004
	Össur	248.906	<0.001	0.998	<0.001	<0.001	<0.001	0.956	<0.001	<0.001
	SACH	138.366	<0.001	0.963	0.038	<0.001	<0.001	<0.001	<0.001	<0.001
	SACH L	358.381	<0.001	0.985	<0.001	<0.001	<0.001	<0.001	<0.001	<0.001
**Mid-Stance**	CP	684.726	<0.001	0.992	<0.001	<0.001	<0.001	<0.001	<0.001	<0.001
	FI	370.003	<0.001	0.986	<0.001	<0.001	<0.001	<0.001	<0.001	0.622
	Össur	576.877	<0.001	0.991	<0.001	<0.001	<0.001	<0.001	<0.001	<0.001
	SACH	1569.185	<0.001	0.997	<0.001	<0.001	<0.001	<0.001	<0.001	<0.001
	SACH L	1225.762	<0.001	0.996	<0.001	<0.001	<0.001	<0.001	<0.001	<0.001
**Terminal Stance**	CP	500.145	<0.001	0.989	<0.001	<0.001	<0.001	<0.001	<0.001	<0.001
	FI	637.239	<0.001	0.992	<0.001	<0.001	<0.001	<0.001	<0.001	<0.001
	Össur	1404.363	<0.001	0.996	<0.001	<0.001	<0.001	<0.001	<0.001	<0.001
	SACH	506.698	<0.001	0.990	<0.001	<0.001	<0.001	<0.001	<0.001	<0.001
	SACH L	188.348	<0.001	0.972	<0.001	<0.001	0.002	<0.001	<0.001	<0.001
**Percent Energy Return**										
**Initial Contact**	CP	86.399	<0.001	0.942	<0.001	<0.001	<0.001	1.000	0.215	0.217
	FI	144.116	<0.001	0.964	<0.001	<0.001	<0.001	0.446	<0.001	<0.001
	Össur	315.599	<0.001	0.983	<0.001	<0.001	<0.001	<0.001	<0.001	0.116
	SACH	412.632	<0.001	0.987	<0.001	<0.001	<0.001	0.015	<0.001	0.198
	SACH L	454.029	<0.001	0.988	<0.001	<0.001	<0.001	0.018	<0.001	0.212
**Mid-Stance**	CP	86.399	<0.001	0.942	<0.001	<0.001	<0.001	0.001	0.927	0.004
	FI	144.116	<0.001	0.964	<0.001	<0.001	<0.001	<0.001	<0.001	0.318
	Össur	315.599	<0.001	0.983	<0.001	<0.001	<0.001	0.417	0.008	<0.001
	SACH	412.632	<0.001	0.987	<0.001	<0.001	<0.001	<0.001	<0.001	0.081
	SACH L	454.029	<0.001	0.988	<0.001	<0.001	<0.001	<0.001	<0.001	0.082
**Terminal Stance**	CP	86.399	<0.001	0.942	<0.001	<0.001	0.012	<0.001	<0.001	<0.001
	FI	144.116	<0.001	0.964	0.037	0.014	<0.001	0.962	<0.001	<0.001
	Össur	315.599	<0.001	0.983	<0.001	<0.001	<0.001	0.088	<0.001	<0.001
	SACH	412.632	<0.001	0.987	0.001	0.053	0.007	0.275	0.830	0.736
	SACH L	454.029	<0.001	0.988	<0.001	<0.001	<0.001	0.853	0.983	0.971

CP = College Park; FI = Freedom Innovations; SACH L = barefoot condition with L-block.

**Table 5:** Statistical results to assess main effect of prosthesis.

	Footwear	F value	p value	η2	Post-hoc									
					CP	CP	CP	CP	FI	FI	FI	Össur	Össur	SACH
					FI	Össur	SACH	SACH L	Össur	SACH	SACH L	SACH	SACH L	SACH L
**Maximum Displacement**														
**Initial Contact**	Bare	2294.38	<0.001	0.998	<0.001	<0.001	<0.001	<0.001	<0.001	<0.001	<0.001	<0.001	0.596	<0.001
	Flat	1130.84	<0.001	0.995	<0.001	<0.001	<0.001	-	<0.001	<0.001	-	<0.001	-	-
	Trainer	242.94	<0.001	0.979	<0.001	<0.001	<0.001	-	<0.001	<0.001	-	0.097	-	-
	Heel	577.71	<0.001	0.991	<0.001	<0.001	<0.001	-	1.000	0.082	-	0.098	-	-
**Mid-** **Stance**	Bare	11748.26	<0.001	1.000	0.996	<0.001	<0.001	<0.001	<0.001	<0.001	<0.001	<0.001	<0.001	<0.001
	Flat	237.34	<0.001	0.978	0.770	<0.001	0.129	-	<0.001	0.529	-	<0.001	-	-
	Trainer	153.88	<0.001	0.967	0.009	<0.001	<0.001	-	<0.001	<0.001	-	<0.001	-	-
	Heel	43.29	<0.001	0.890	<0.001	<0.001	<0.001	-	0.681	<0.001	-	0.004	-	-
**Terminal Stance**	Bare	4694.57	<0.001	0.999	<0.001	<0.001	<0.001	<0.001	<0.001	<0.001	<0.001	<0.001	<0.001	<0.001
	Flat	2419.01	<0.001	0.998	<0.001	<0.001	<0.001	-	<0.001	<0.001	-	<0.001	-	-
	Trainer	1154.62	<0.001	0.995	<0.001	<0.001	<0.001	-	<0.001	<0.001	-	<0.001	-	-
	Heel	2458.01	<0.001	0.998	<0.001	<0.001	<0.001	-	<0.001	<0.001	-	<0.001	-	-
**Percent Energy Return**														
**Initial Contact**	Bare	807.26	<0.001	0.994	<0.001	<0.001	<0.001	<0.001	<0.001	<0.001	<0.001	<0.001	<0.001	0.039
	Flat	422.41	<0.001	0.988	0.091	<0.001	<0.001	-	<0.001	<0.001	-	<0.001	-	-
	Trainer	141.50	<0.001	0.964	0.011	<0.001	<0.001	-	<0.001	0.002	-	<0.001	-	-
	Heel	323.82	<0.001	0.984	<0.001	<0.001	<0.001	-	<0.001	<0.001	-	<0.001	-	-
**Mid-** **Stance**	Bare	423.65	<0.001	0.988	<0.001	<0.001	0.014	<0.001	<0.001	0.709	<0.001	<0.001	<0.001	<0.001
	Flat	102.56	<0.001	0.951	0.482	<0.001	<0.001	-	<0.001	0.013	-	<0.001	-	-
	Trainer	98.03	<0.001	0.948	0.001	<0.001	0.063	-	<0.001	0.243	-	<0.001	-	-
	Heel	65.65	<0.001	0.925	0.072	<0.001	0.001	-	<0.001	0.102	-	<0.001	-	-
**Terminal Stance**	Bare	115.18	<0.001	0.958	0.010	1.000	<0.001	<0.001	0.008	0.044	<0.001	<0.001	<0.001	<0.001
	Flat	88.42	<0.001	0.943	0.730	0.197	<0.001	-	0.029	<0.001	-	<0.001	-	-
	Trainer	107.16	<0.001	0.953	<0.001	<0.001	0.996	-	<0.001	0.001	-	<0.001	-	-
	Heel	417.29	<0.001	0.987	0.962	0.121	<0.001	-	0.266	<0.001	-	<0.001	-	-

CP = College Park; FI = Freedom Innovations; SACH L = barefoot condition with L-block.

In support of our hypothesis and in agreement with prior work [8], the addition of footwear to prosthetic feet that account for different heel heights generally increased the maximum displacement (deformation) and decreased the percentage of energy return (energy efficiency) when compared to barefoot. Although deformation increased with footwear for every prosthesis for initial contact loading, the flat shoe was the only footwear condition to decrease maximum displacement for terminal stance loading and was likely due to the specific orientation of the prosthesis when aligned as this shoe was of minimal material. Differences in displacement among footwear conditions at midstance were relatively small, as was the absolute displacement relative to initial contact and terminal stance, likely due to contact, and hence loading, of both the heel and keel regions of the prostheses with the loading surface. These small displacements at midstance observed for every prosthesis-footwear combination translated to greater stiffness (Table 4), suggesting minimal vertical travel when the prosthesis is loaded with an upright pylon. Qualitatively, while the addition of footwear often tended to converge behavior across prostheses to similar levels of maximum displacement at initial contact and midstance, the prosthetic feet retained their unique deformation characteristics at terminal stance (Fig 4). Consequently, prosthetists and users of these prosthetic feet can expect similar trends when adding footwear accommodated by these feet, and this seems to hold for the SACH feet despite the fact that different devices were used for each footwear condition. However, notably, the more rigid SACH feet demonstrated considerably less deformation at terminal stance (48% average reduction across shod conditions) compared to the other prostheses (individual differences all being statistically significant, Table 5) that are essentially dynamic response feet.

During initial contact and terminal stance, prosthetic deformation can simulate plantarflexion and dorsiflexion, respectively [6, 18]. While greater deformation could be perceived as easier advancement over the third rocker at terminal stance and a more stable limb position during initial loading, extreme deformation can translate to a drop off effect [19] and sinking into the prosthesis, respectively. For users of prostheses with ankle adjustment to account for footwear of different heel heights, these sensations would need to be adjusted to when quickly swapping shoes. However, recent evidence suggests that women prosthesis users can very quickly adapt to changes in footwear when walking with user-controlled adjustable prosthetic feet [20]. It is important to note that deformation of the prosthetic feet are not only a function of the additional footwear, but also the alignment of the prosthesis [14]. For example, alignment likely played a key role in the relatively low initial contact displacement values in the trainer, despite a relatively soft sole. This dual contribution is clearly evident for the SACH foot with 0 cm rise, which was tested barefoot with and without the L-block. The slight rise of the heel with the L-block (10 mm) reduced displacement from 17.1 to 14.6 mm (18% decrease) for initial contact and increased displacement from 9.0 to 12.1 mm (34% increase) for terminal stance, both differences being significant (Table 5). Therefore, even small adjustments in the angular positioning of the prosthesis relative to the pylon altered its mechanical response as different regions of the foot were engaged during loading. This observation emphasizes why it is important to mechanically characterize prostheses independent of the user but under varied loading conditions (orientations, magnitude, rate) through standardized methods for a more universal comparison, as users will apply their individual gait mechanics to load the prosthetic system in different ways and thereby experience a unique response. The estimated changes in stiffness due to footwear and realignment appear to be within the range that would alter prosthetic ankle-foot kinematics [18, 21] and can be perceived by transtibial prosthesis users [3]. However, irrespective of the change in prosthesis mechanical response, walking with a 5.08 cm heel will certainly require considerable modifications to overall gait dynamics when compared to wearing a trainer or flat shoe [22–26] and the application of these results should be interpreted with this in mind.

The ability of the prosthesis to store and dissipate energy at initial contact is relevant to its function in saving the residual limb from assuming that energy during limb collision that could lead to tissue damage [2]. Although there were notable differences in energy storage in the barefoot condition (S1 Table), all prosthetic feet tended to absorb similar levels when shod apart from the College Park foot, which consistently stored less energy. Given this foot was also the stiffest in that loading scenario (Table 3), the prosthesis will deform less under the same load (Fig 4) to store less energy. Despite different levels in energy storage, the addition of footwear consistently decreased the percentage of energy returned (Fig 5). However, even with the considerable changes in footwear design and prosthesis alignment, the percentage of energy return at initial contact was similar across shod conditions for each individual prosthetic foot.

The ability of the prosthesis to store and dissipate energy for minimizing energy transfer to the residual limb in initial contact also extends to midstance [2], where again the addition of footwear consistently increased the amount of energy stored and decreased the percentage of energy returned (relative to energy stored) (Fig 5). Notably, the flat shoe had the greatest effect on reducing energy efficiency. Because this shoe was of minimal material, that reduction compared to the other shod conditions is likely a reflection of changes in alignment (or prosthesis in the case of the SACH foot) as the heel is dropped to the level of the forefoot when aligned. Energy storage and return during midstance may be of lesser importance to the user experience than during loading and push-off, but midstance properties may contribute, in part, to the comfort of loading when stepping down from a curb for instance when the prosthesis can absorb/dissipate more energy.

In terminal stance, energy return is relevant to supporting the transition of the limb from stance to swing through propulsion when timed correctly. As passive-elastic feet do not generate energy, propulsive capability is dependent on the amount of energy returned in terminal stance. Although the absolute amount of energy returned at terminal stance varied across prosthetic feet with the addition of footwear (S2 Table), the energy efficiency of the adjustable heel height prosthetic feet were similar across shod conditions apart from the SACH foot, which consistently returned less energy relative to the amount it stored (Fig 5) compared to the other feet (all differences being significant, Table 5). Moreover, similar to the displacement results, lifting the heel of the SACH foot by 10 mm when using the L-block significantly reduced the percentage of energy return from 73% to 55%, suggesting a more plantarflexed alignment can enhance energy dissipation and result in slightly less plantar contact (reduced lever arm) during loading in the terminal stance orientation. Importantly, the energy efficiency of the adjustable feet was similar to other dynamic response feet [8, 27], suggesting that performance is likely not compromised with user-controlled, adjustable heel-height prosthetic feet. Importantly, compared to other footwear, use of the flat shoe appeared to have a more pronounced but varied effect on maximum displacement (Fig 4, Table 4) than energy return (Fig 5, Table 4). Combined, these energy efficiency results suggest that, at least for common footwear, prosthetists and prosthesis users can expect similar energy efficiency performance for a respective prosthetic foot when swapping shoes and adjusting the prosthesis accordingly.

The characterization results from this study add to limited information on the mechanical function of commercial prosthetic feet designed to accommodate footwear of different heel heights. Although men also wear shoes of different heel heights, the footwear included in this study are particularly relevant to women prosthesis users. Overall, results suggest the addition of these types of footwear can affect the mechanical function of the prostheses studied. Importantly, those changes are a function of both the footwear and alignment of the foot relative to the proximal pylon. While the effects of alignment alone were observed in the SACH foot, a limitation of this study is that those independent alignment effects were not characterized for every prosthesis as that did not reflect the mechanism by which they adjust to footwear (i.e., via ankle rotation). Generally, prosthetists should be mindful that the use of prosthetic feet that allow for small user-controlled adjustments in sagittal plane could in fact impact the way the prosthesis responds to weight bearing and hence also the user experience. It may be prudent for patients to receive education on how such changes in footwear and alignment may affect prosthesis function, but perhaps with greater emphasis on prosthesis deformation and stiffness rather than energy efficiency as that property was less impacted. Future work should focus on characterizing the relationships between the changes in mechanical function of heel height adjustable prosthetic feet and clinically-relevant user outcomes. Knowledge of prosthesis mechanical function through standardized bench testing as employed in this study coupled with associated rehabilitation outcomes will continue to help refine prescription guidelines for women prosthesis users.

An important note on results interpretation is that the measurements reported in this study are specific to the mechanical characterization method and associated features (loading orientation, range, and rate as recommend by the American Prosthetics and Orthotics Association [16]) and so results are best interpreted as a relative within-study measure. Furthermore, these characterizations were performed over a larger compressive force range than might be experienced by a 68–70 kg user during normal walking without carrying load. As such, raw data are available open-access to address additional reader-specific questions. Additionally, while linear stiffness approximations are an oversimplification of the prosthesis behavior and were presented as a more interpretable measure, more accurate modeling to capture full behavior can be applied. Finally, as is common with bench test methods of the type used here [8, 16], the loading rate is lower than that experienced during walking and this limitation should be considered. Future work may consider more universal methods (apparatus plus analysis) for characterization that allow for broader comparison across prostheses, such as that undertaken by the International Standards Organization [28].

## Conclusion

The aim of this study was to characterize the effects of women-specific footwear on the mechanical function of commercial prosthetic feet that can accommodate to different heel heights. The results suggest that on average the addition of footwear increases displacement under load and decreases the percentage of energy returned compared to barefoot. However, while including a flat, trainer and 5.08 cm heel shoe had varying effects on absolute deformation, they produced similar levels of energy efficiency. Importantly, the observed changes in mechanical function in this study were a function of both adding footwear and adjustments in alignment to accommodate different heel heights. Prosthetists and prosthesis users who select to wear this footwear with heel height adjustable prosthetic feet should be aware of potential changes in mechanical function that could impact the user experience.

## Supporting information

S1 Table Energy stored. Energy stored (Joules; mean [95% confidence interval]). CP = College Park; FI = Freedom Innovations; SACH L = barefoot condition with L-block.
(DOCX)

S2 Table Energy returned. Energy returned (Joules; mean [95% confidence interval]). CP = College Park; FI = Freedom Innovations; SACH L = barefoot condition with L-block.
(DOCX)

S1 Fig Initial contact hysteresis curves. Representative force-displacement curves for initial contact loading.
(DOCX)

S2 Fig Midstance hysteresis curves. Representative force-displacement curves for midstance loading.
(DOCX)

S3 Fig Terminal stance hysteresis curves. Representative force-displacement curves for terminal stance loading.
(DOCX)
