# Supplementary material for: Effects of women’s footwear on the mechanical function of heel-height accommodating prosthetic feet
Source: PLoS One. 2022 Jan 24;17(1):e0262910. doi: 10.1371/journal.pone.0262910 (PMC8786192; doi:10.1371/journal.pone.0262910)
Supplement: S1 Table — Energy stored (Joules; mean [95% confidence interval]). CP = College Park; FI = Freedom Innovations; SACH L = barefoot condition with L-block. (DOCX) [file pone.0262910.s001.docx]

**S1 Table.** Energy stored (Joules; mean [95% confidence interval]). CP=College Park; FI=Freedom Innovations; SACH L=barefoot condition with L-block.

|  |  | Barefoot | Flat | Trainer | Heel |
| --- | --- | --- | --- | --- | --- |
| Initial contact | CP | 5.3 [0.1] | 6.8 [0.1] | 9.4 [0.2] | 6.9 [0.1] |
|  | FI | 7.6 [0.1] | 10.8 [0.1] | 10.6 [0.2] | 10.7 [0.2] |
|  | Össur | 7.0 [0.0] | 11.3 [0.1] | 11.2 [0.1] | 10.3 [0.1] |
|  | SACH | 9.6 [0.1] | 10.9 [0.2] | 11.5 [0.2] | 11.2 [0.1] |
|  | SACH L | 8.1 [0.1] | NA | NA | NA |
| Midstance | CP | 2.8 [0.0] | 3.1 [0.0] | 4.1 [0.1] | 3.4 [0.1] |
|  | FI | 2.5 [0.0] | 3.0 [0.1] | 3.7 [0.1] | 3.5 [0.1] |
|  | Össur | 3.6 [0.0] | 3.9 [0.0] | 4.8 [0.1] | 3.7 [0.0] |
|  | SACH | 1.4 [0.0] | 2.8 [0.1] | 4.1 [0.1] | 3.6 [0.1] |
|  | SACH L | 1.6 [0.0] | NA | NA | NA |
| Terminal stance | CP | 12.7 [0.1] | 11.5 [0.1] | 14.2 [0.4] | 16.0 [0.4] |
|  | FI | 12.4 [0.2] | 10.4 [0.1] | 12.2 [0.0] | 12.2 [0.1] |
|  | Össur | 16.8 [0.1] | 12.6 [0.2] | 13.5 [0.2] | 14.4 [0.1] |
|  | SACH | 4.6 [0.1] | 4.6 [0.1] | 8.9 [0.1] | 6.9 [0.2] |
|  | SACH L | 5.3 [0.2] | NA | NA | NA |
