# Supplementary material for: Effects of women’s footwear on the mechanical function of heel-height accommodating prosthetic feet
Source: PLoS One. 2022 Jan 24;17(1):e0262910. doi: 10.1371/journal.pone.0262910 (PMC8786192; doi:10.1371/journal.pone.0262910)
Supplement: S2 Table — Energy returned (Joules; mean [95% confidence interval]). CP = College Park; FI = Freedom Innovations; SACH L = barefoot condition with L-block. (DOCX) [file pone.0262910.s002.docx]

**Table S2.** Energy returned (Joules; mean [95% confidence interval]). CP=College Park; FI=Freedom Innovations; SACH L=barefoot condition with L-block.

|  |  | Barefoot | Flat | Trainer | Heel |
| --- | --- | --- | --- | --- | --- |
| Initial contact | CP | 4.1 [0.0] | 4.7 [0.0] | 6.6 [0.0] | 4.9 [0.0] |
|  | FI | 6.0 [0.0] | 7.6 [0.0] | 7.6 [0.0] | 7.3 [0.1] |
|  | Össur | 6.4 [0.0] | 9.8 [0.0] | 9.0 [0.0] | 8.4 [0.1] |
|  | SACH | 8.4 [0.1] | 8.2 [0.1] | 8.6 [0.1] | 8.2 [0.0] |
|  | SACH L | 7.1 [0.1] | NA | NA | NA |
| Midstance | CP | 2.4 [0.0] | 2.3 [0.0] | 3.2 [0.0] | 2.6 [0.0] |
|  | FI | 2.1 [0.0] | 2.2 [0.0] | 2.8 [0.0] | 2.7 [0.0] |
|  | Össur | 3.3 [0.0] | 3.3 [0.0] | 3.9 [0.0] | 3.1 [0.0] |
|  | SACH | 1.1 [0.0] | 2.0 [0.0] | 3.2 [0.0] | 2.8 [0.0] |
|  | SACH L | 1.4 [0.0] | NA | NA | NA |
| Terminal stance | CP | 10.7 [0.1] | 8.9 [0.1] | 11.3 [0.2] | 13.1 [0.3] |
|  | FI | 9.7 [0.2] | 8.0 [0.0] | 9.3 [0.1] | 10.0 [0.1] |
|  | Össur | 14.1 [0.1] | 9.9 [0.0] | 10.7 [0.1] | 11.7 [0.1] |
|  | SACH | 3.2 [0.0] | 3.2 [0.0] | 6.3 [0.0] | 4.8 [0.1] |
|  | SACH L | 2.9 [0.3] | NA | NA | NA |
