# Supplementary figures and images for: Effects of women’s footwear on the mechanical function of heel-height accommodating prosthetic feet
Source: PLoS One. 2022 Jan 24;17(1):e0262910. doi: 10.1371/journal.pone.0262910 (PMC8786192; doi:10.1371/journal.pone.0262910)

**
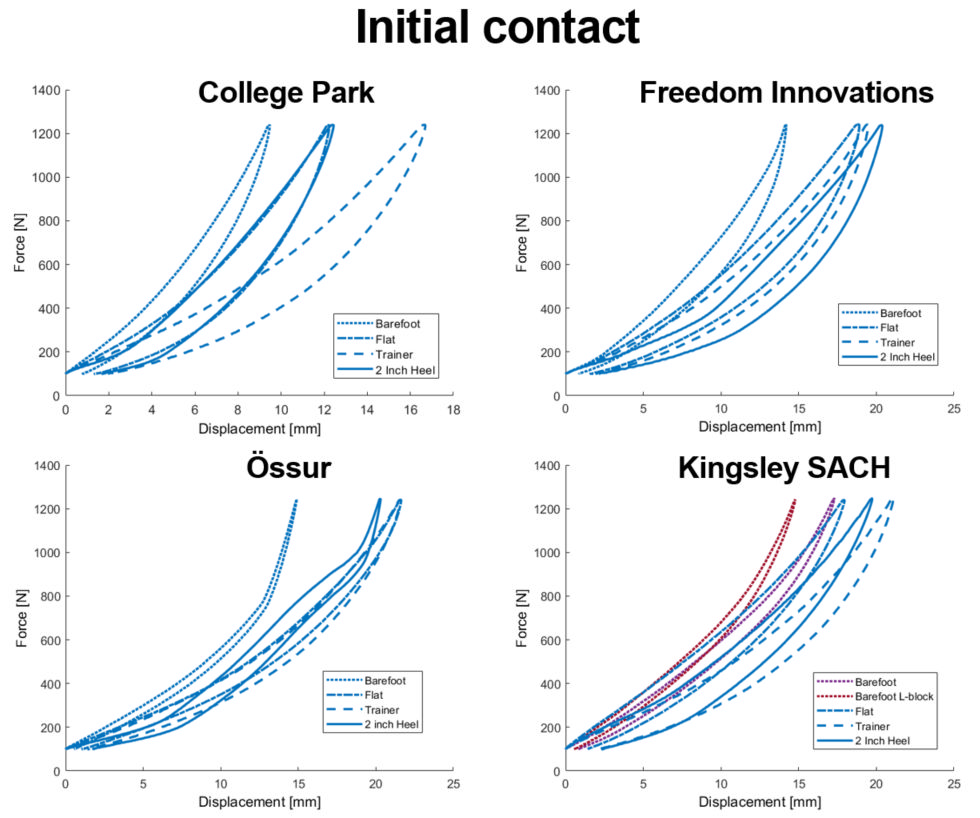
**
**S1 Figure.** Representative force-displacement curves for initial contact loading.

Supplement: S1 Fig — Representative force-displacement curves for initial contact loading. (DOCX) [file pone.0262910.s003.docx]

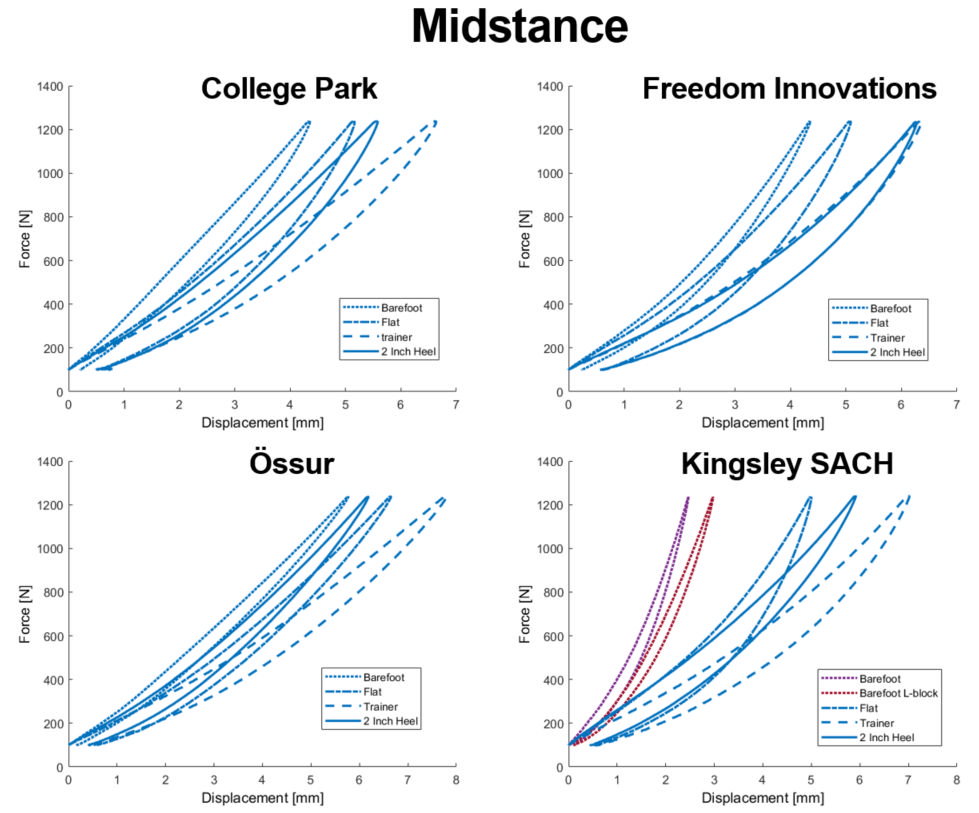


**S2 Figure.** Representative force-displacement curves for midstance loading.

Supplement: S2 Fig — Representative force-displacement curves for midstance loading. (DOCX) [file pone.0262910.s004.docx]

**
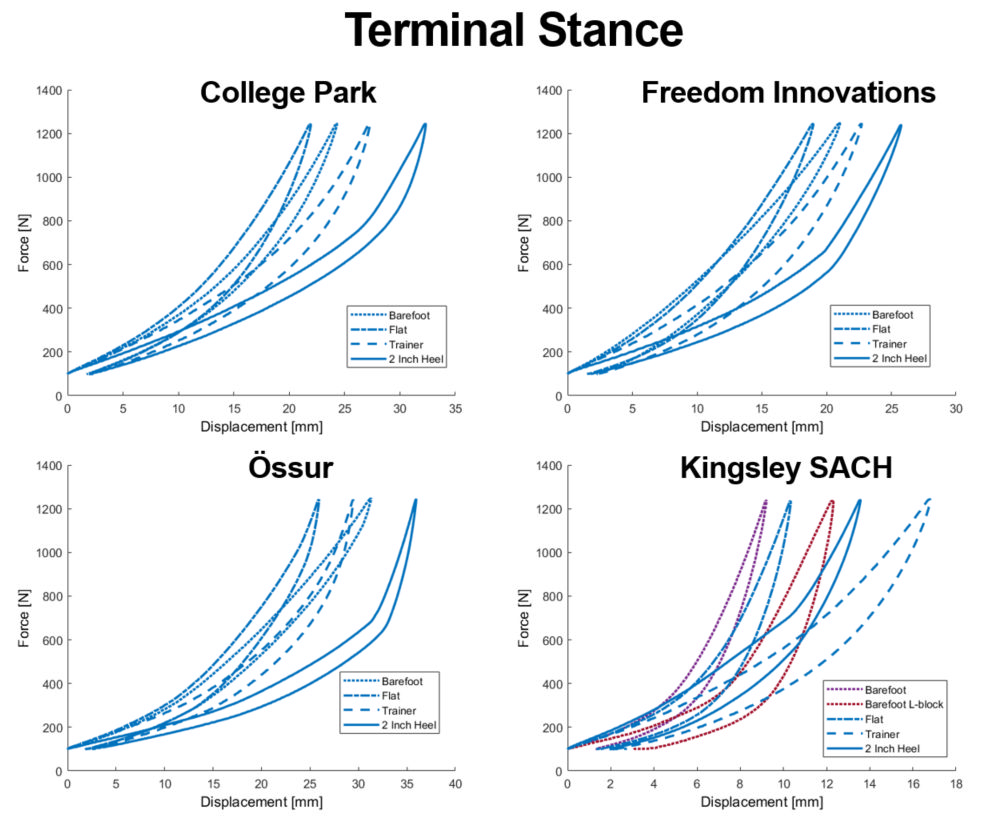
**

**S3 Figure**. Representative force-displacement curves for terminal stance loading.

Supplement: S3 Fig — Representative force-displacement curves for terminal stance loading. (DOCX) [file pone.0262910.s005.docx]
